# Leaf Venation and Morphology Help Explain Physiological Variation in *Yucca brevifolia* and *Hesperoyucca whipplei* Across Microhabitats in the Mojave Desert, CA

**DOI:** 10.3389/fpls.2020.578338

**Published:** 2021-01-08

**Authors:** Amber R. Jolly, Joseph Zailaa, Ugbad Farah, Janty Woojuh, Félicia Makaya Libifani, Darlene Arzate, Christian Alex Caranto, Zayra Correa, Jose Cuba, Josephina Diaz Calderon, Nancy Garcia, Laura Gastelum, Ivette Gutierrez, Matthew Haro, Monserrat Orozco, Jessica Lamban Pinlac, Andoni Miranda, Justin Nava, Christina Nguyen, Edgar Pedroza, Jennyfer Perdomo, Scott Pezzini, Ho Yuen, Christine Scoffoni

**Affiliations:** Department of Biological Sciences, California State University, Los Angeles, Los Angeles, CA, United States

**Keywords:** 3D venation, chaparral yucca, Joshua tree, LMA, monocots, succulence

## Abstract

Different microclimates can have significant impact on the physiology of succulents that inhabit arid environments such as the Mojave Desert (California). We investigated variation in leaf physiology, morphology and anatomy of two dominant Mojave Desert monocots, *Yucca brevifolia* (Joshua tree) and *Hesperoyucca whipplei*, growing along a soil water availability gradient. Stomatal conductance (*g*_s_) and leaf thickness were recorded in the field at three different sites (north-western slope, south-eastern slope, and alluvial fan) in March of 2019. We sampled leaves from three individuals per site per species and measured in the lab relative water content at the time of *g*_s_ measurements, saturated water content, cuticular conductance, leaf morphological traits (leaf area and length, leaf mass per area, % loss of thickness in the field and in dried leaves), and leaf venation. We found species varied in their *g*_s_: while *Y. brevifolia* showed significantly higher *g*_s_ in the alluvial fan than in the slopes, *H. whipplei* was highest in the south-eastern slope. The differences in *g*_s_ did not relate to differences in leaf water content, but rather to variation in number of veins per mm^2^ in *H. whipplei* and leaf width in *Y. brevifolia*. Our results indicate that *H. whipplei* displays a higher water conservation strategy than *Y. brevifolia*. We discuss these differences and trends with water availability in relation to species’ plasticity in morphology and anatomy and the ecological consequences of differences in 3-dimensional venation architecture in these two species.

## Introduction

Plants have different physiological thresholds to survive drought and understanding these thresholds can help predict community structure as climate change persists (Choat et al., 2018). The structure and function of plant populations are greatly determined by the environment, especially in deserts, a quickly growing biome (Cloudsley-Thompson, 1978; Archer and Predick, 2008) where temperature and moisture limitations fluctuate drastically (St. Clair and Hoines, 2018). Plants native to arid ecosystems have adapted special anatomical, morphological and physiological traits, enabling them to survive and thrive under conditions of water scarcity and extreme temperatures. The Mojave Desert is dominated by winter precipitation making it an ideal home for the iconic *Yucca brevifolia*, commonly known as the Joshua tree (Archer and Predick, 2008). However, the standing groves of the *Y. brevifolia* and another important Mojave Desert succulent, *Hesperoyucca whipplei*, are threatened because of the ongoing major migration of their ideal habitats due to climate change (Wells and Woodcock, 1985; Cole et al., 2011).

*Y. brevifolia* is a C3 monocotyledonous evergreen species endemic to the Mojave Desert of southwest California, Nevada, Utah and Arizona. It typically resides at the elevations ranging from 1,000 to 2,000 m (Brittingham and Walker, 2000). *Y. brevifolia* grows on average 3.7 cm per year and has a median life expectancy of about 90 years (Gilliland et al., 2006), but little is known of the physiology of this species. Previous studies investigated rosette and flower panicle orientations and found that both were predominately oriented toward the south to maximize solar radiation (Warren et al., 2016) and these south facing rosettes tend to have higher stomatal conductance (Rasmuson et al., 1994).

However, a lack of knowledge still exists of the hydraulic properties of this species and how these relate to anatomy. *Y. brevifolia* has a discontinuous distribution in the Mojave Desert, with highest population density on well-drained alluvial fans adjacent to desert mountain ranges (Cole et al., 2011). Because of this narrow range of abiotic climate conditions, *Y. brevifolia* populations have been shifting to higher elevations with climate change (Harrower and Gilbert, 2018). Indeed, the elevated temperatures consequent to climate change result in shorter frost seasons, and thus, seasonal water availability, causing the reduction and range shift of *Y. brevifolia* populations (Archer and Predick, 2008). Differences in shifts occurs throughout the entire range of this species. Indeed, the loss of the distribution of *Y. brevifolia* has been suggested in primarily its southern and central ranges, highlighting potential habitat expansion in the north and east though the occupation of these new areas are noted to be unlikely to happen via natural seed dispersal (Cole et al., 2011). Additionally, the northern range expansion could be limited by factors such as invasive grasses which may intensify wildfires (Sweet et al., 2019). Increase in fires in the northern range could further limit *Y. brevifolia*’s expansion there, as a study at the southern range found that increased fires due to prolonged droughts increased mortality of *Y. brevifolia*, especially in the younger individuals (Defalco et al., 2010). Finally, the distribution of the mutualistic yucca moths (*Tegeticula synthetica* and *Tegeticula antithetica*) must match the expansion into refugia regions (Sweet et al., 2019), which is dependent on the elevation gradient (Harrower and Gilbert, 2018). A finer scale study, focused on the southern range of *Y. brevifolia* found that under a 3°C temperature increase scenario, 90% of the present distribution would be reduced (Barrows and Murphy-Mariscal, 2012). Warming temperatures also negatively impacted seed germination (Keeley and Meyers, 1985), which could further exacerbate the population decline of this species with climate change. These alarming findings point to the urge to better characterize the physiology and anatomy of this species in hopes to better understand how it might acclimate to warmer and drier environments with climate change. Here, we explore physiological and anatomical variation in *Y. brevifolia* across microhabitats varying in soil composition and moisture.

*Hesperoyucca whipplei*, commonly known as the chaparral yucca, is a C3 species more widely distributed than *Y. brevifolia. H*. *whipplei* extends north from San Diego into the Mojave Desert of Southern California and is discontinuous throughout its range (Haines, 1941; Aker, 1982; Davis et al., 1994). Contrary to *Y. brevifolia*, *H. whipplei* populations usually occur in shallow soil or rocky outcrops and inhabit elevations from sea level up to 2,450 m (Haines, 1941). Little is known about the hydraulic properties of *H. whipplei* and how this species may respond to a changing climate. Previous work has mapped the historic extant of *H. whipplei* in the Death Valley region and found that there is evidence that its distribution has diminished over time there possibly due the increase in summer temperature and aridity (Wells and Woodcock, 1985). However, a study comparing seedlings of *Y. brevifolia* and *H. whipplei* showed that when grown under elevated CO_2_, *H. whipplei* was able to maintain photosynthesis despite a decline in *g*_s_ at high temperatures, whereas *Y. brevifolia* was more sensitive to high temperatures, experiencing a decline in both photosynthesis and *g*_s_ (Huxman et al., 1998). These results suggest that *H. whipplei* might have greater potential to acclimate to climate change. But these differences in physiology were never investigated in the field across microhabitats varying in soil moisture.

We investigated the leaf physiological, morphological, and anatomical properties of these two ecologically important desert succulents, *Y. brevifolia* and *H. whipplei*, growing in three different microhabitats in the West Mojave Desert: a north-western slope, south-eastern slope, and an alluvial fan. Alluvial fans are known to have deep, sandy soils with high moisture and nutrient levels, while water run-off areas such as slopes consist of shallow, rocky soils with poor nutrient levels and water retention (Brooks, 1999). We hypothesize that the highest *g*_s_ would be observed at sites most representative of the species preferred habitat: *Y. brevifolia* at the alluvial fan and *H. whipplei* at the south-eastern slope (i.e., the site with least water and nutrient retention as a result of soil topography and the increased potential evapotranspiration due to the sun’s diurnal trajectory). We also expect these physiological differences to be constrained by morpho-anatomical traits across sites, with individuals with lower vein density and thinner leaves displaying lower *g*_s_ values.

## Materials and Methods

### Study Species and Site Description

**FIGURE 1:**
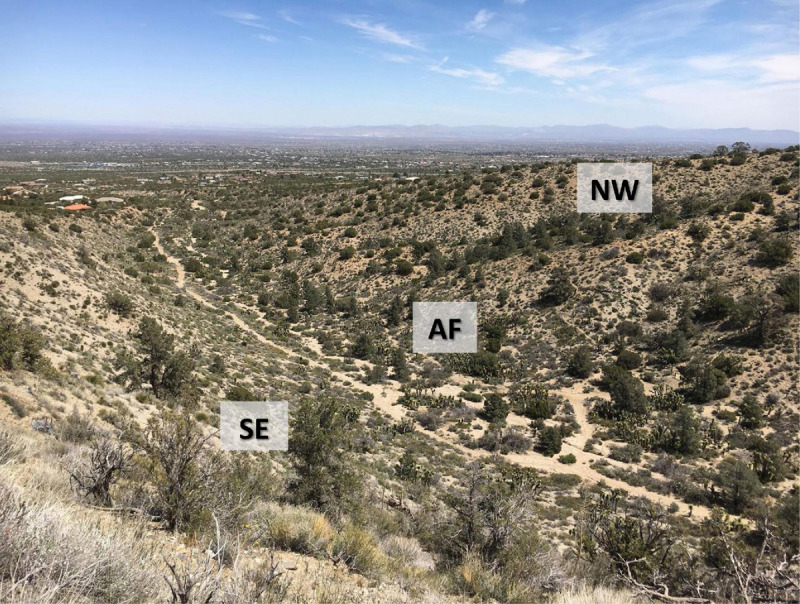
Study sites in the West Mojave Desert. Overview of the three sites (NW, north-western slope; AF, alluvial fan; SE, south-eastern slope) in Puma Canyon in Piñon Hills, California. *n* = 3–8 individuals per species per site.

Data was collected in March of 2019 (March 10, 15, and 24) () on two desert succulent species (*Yucca brevifolia* and *Hesperoyucca whipplei*) from at the Puma Canyon Ecological Reserve (34°24′22.6′′N 117°37′11.3′′W), from three sites differing in soil moisture and composition (*n* = 3–8 individuals per species per site; Table 1). Our sites consisted of a north-western slope (NW) and a south-eastern slope (SE) exhibiting rocky soils, separated by an alluvial fan with sandy soil (AF) (Figure 1). Elevation across sites ranged from 1,300 to 1,400 m. To characterize the environmental conditions in the three microhabitats, we measured air temperature and vapor-pressure deficit (VPD) throughout the days of measurement with a Delta-T AP4 Leaf Porometer (Dynamax, TX, United States). We quantified differences in soil moisture at 20–30 cm below the surface with a soil moisture meter at six different locations per site, with three measurements per locations (dry/wet index from 1–10; Dr. meter Hygrometer Moisture Sensor, Taiwan). Notably, no variation in either air temperature or VPD was recorded across sites during sampling days (Table 1). Soil moisture significantly varied across sites, with the alluvial fan being significantly moister than the south-eastern slope (Table 1).

**TABLE 1:** Environmental conditions (air temperature, soil moisture, and vapor-pressure deficit) at the three sites in Puma Canyon Ecological Reserve.

	**Air temperature (°C)**	**Soil moisture (1–10 index)**	**Vapor-pressure deficit (kPa)**
South-eastern slope	6.64 ± 0.21 (a)	2.31 ± 0.13 (b)	1.11 ± 0.008 (a)
Alluvial fan	7.19 ± 0.27 (a)	3.22 ± 0.36 (a)	1.12 ± 0.009 (a)
North-western slope	6.76 ± 0.17 (a)	2.31 ± 0.22 (ab)	1.12 ± 0.009 (a)
One-way ANOVA (site)	*p* = 0.751	*p* = 0.019	*p* = 0.999

*Letters in parenthesis indicate significant differences across means at *p* = 0.05 (*n* = 9–49).*

### Physiological Traits

Individuals from *Y. brevifolia* and *H. whipplei* were chosen 2 and 1 m apart, respectively, to avoid sampling clonal sprouts. Stomatal conductance (*g*_s_) was measured in the field between approximately 10:00 and 15:00 h on the abaxial surface of 3–5 leaves from each of 6–8 individuals per species and site using a Delta-T AP4 Leaf Porometer (Dynamax, TX, United States). We tested whether *g*_s_ measurements were influenced by the time of measurement and by the VPD at the time of measurement by plotting *g*_s_ against each one of these variables at each site for each species (Supplementary Figures S1, S2). We found no significant correlations, except for *H. whipplei* that displayed a negative correlation between time and *g*_s_ in the AF, and a positive correlation between *g*_s_ and VPD in the AF and SE slope. Notably, the *g*_s_ measurements taken at a later time of day were also those with very low VPD. Such conditions would likely induce stomatal closure. We thus decided to remove those data points from the analysis. We note that our results were not affected by the inclusion or exclusion of these points.

We collected 21 leaves (three leaves from five individuals; including some for which we had measured *g*_s_ on) per species per site from the top third of the rosette and rapidly placed them in a Whirlpak bag (Nasco, GA, United States). Bags were exhaled into to increase relative humidity and CO_2_ concentration, causing stomata to close and preventing further water loss from the leaf samples. Individual bags were then placed in large black plastic bags filled with moist paper towels to further prevent sample dehydration, placed in a cooler and transported to the laboratory within a few hours. Once in the lab, 15 leaves from three individuals were removed from the bags and we measured both leaf mass using an analytical balance (±10 μg; model XS205; Mettler Toledo) and leaf thickness (using calipers; ±0.01 mm; Fowler). These leaves and remaining samples were then rehydrated overnight in beakers with ultrapure water, covered in plastic bags filled with wet paper towels to ensure high relative humidity around the rehydrating leaves. From these rehydrated leaves, the minimum epidermal conductance (*g*_min_) was obtained from six, and the 15 leaves measured for initial mass and thickness were remeasured the next day after rehydration. We placed 10 of these leaves in a drying oven at 75°C, and kept 5 leaves from three individuals for anatomical measurements (see below). After a minimum of 3 days in the oven, leaves were measured for dry mass. We calculated the relative water content for each leaf using Eq. (1):

(1) $$R\,W\,C=\frac{\left(l\,e\,a\,f\,m\,a\,s\,s\,i\,n\,f\,i\,e\,l\,d-l\,e\,a\,f\,d\,r\,y\,m\,a\,s\,s\right)}{r\,e\,h\,y\,d\,r\,a\,t\,e\,d\,l\,e\,a\,f\,m\,a\,s\,s-l\,e\,a\,f\,d\,r\,y\,m\,a\,s\,s}*100$$

Saturated water content was calculated using Eq. (2):

(2) $$S\,W\,C=\frac{d\,r\,y\,l\,e\,a\,f\,m\,a\,s\,s}{r\,e\,h\,y\,d\,r\,a\,t\,e\,d\,l\,e\,a\,f\,m\,a\,s\,s-l\,e\,a\,f\,m\,a\,s\,s\,i\,n\,t\,h\,e\,f\,i\,e\,l\,d}$$

The percent loss thickness (PLT) of leaves collected in the field was calculated using Eq. (3):

(3) $$\text{PLT}=100-\left(a\,v\,e\,r\,a\,g\,e\,i\,n\,i\,t\,i\,a\,l\,t\,h\,i\,c\,k\,n\,e\,s\,s*\frac{100}{a\,v\,e\,r\,a\,g\,e\,r\,e\,h\,y\,d\,r\,a\,t\,e\,d\,t\,h\,i\,c\,k\,n\,e\,s\,s}\right)$$

The minimum epidermal conductance, also referred to as cuticular conductance (*g*_min_) was measured using the “clothesline” method (protocol from Sack and Scoffoni, 2011), based on the decline in mass through vapor diffusion of the leaf epidermis. Two leaves from three individuals per site and species were collected in the field as described above. In the lab, leaves were rehydrated overnight. The following day, *g*_min_ was measured as described in published protocol (Sack and Scoffoni, 2011).

### Leaf Morphological Traits

From the 15 leaves collected described above, maximum thickness was obtained from the middle of each leaf using a caliper (±0.01 mm; Fowler) the day after leaves were rehydrated, along with leaf area and leaf length. Individual leaves were photographed and analyzed for area, width, and length in ImageJ (version 1.52a^1^).

To account for the 3D nature of the leaves, leaf surface area (*n* = 9–10 leaves per species per site) was measured using leaf geometry following previous literature (Huxman et al., 1998), considering 2/3 of the leaf length as a rectangle and 1/3 of the leaf length as a triangle (Supplementary Figure S3). To account for leaf tapering, the rectangle calculation only included leaf thickness and width from the top and middle of the leaves, while the triangle calculation only included the leaf thickness and width from the top. All surfaces of the leaf were considered. Area of the top and bottom of the rectangle (representing the adaxial and abaxial surfaces of the bottom two thirds of the leaf) were calculated as follows:

A⁢r⁢e⁢a⁢⁢o⁢f⁢⁢t⁢o⁢p⁢⁢a⁢n⁢d⁢⁢b⁢o⁢t⁢t⁢o⁢m⁢⁢r⁢e⁢c⁢t⁢a⁢n⁢g⁢l⁢e⁢s

(4) $$=2\,\left(\frac{2}{3}*t\,o\,t\,a\,l\,l\,e\,a\,f\,l\,e\,n\,g\,t\,h*a\,v\,e\,r\,a\,g\,e\,l\,e\,a\,f\,t\,h\,i\,c\,k\,n\,e\,s\,s\right)$$

To measure the area of the left and right sides of the rectangle (representing the sides of the bottom two thirds of the leaf), we used Eq. (5):

A⁢r⁢e⁢a⁢⁢o⁢f⁢⁢l⁢e⁢f⁢t⁢⁢a⁢n⁢d⁢⁢r⁢i⁢g⁢h⁢t⁢⁢r⁢e⁢c⁢t⁢a⁢n⁢g⁢l⁢e⁢s

(5) $$=2\,\left(\frac{2}{3}*t\,o\,t\,a\,l\,l\,e\,a\,f\,l\,e\,n\,g\,t\,h*a\,v\,e\,r\,a\,g\,e\,l\,e\,a\,f\,w\,i\,d\,t\,h\right)$$

To consider all four surfaces of the triangle, the top and bottom (representing the adaxial and abaxial surfaces of the top third of the leaf) were calculated as follows:

A⁢r⁢e⁢a⁢⁢o⁢f⁢⁢t⁢o⁢p⁢⁢a⁢n⁢d⁢⁢b⁢o⁢t⁢t⁢o⁢m⁢⁢r⁢e⁢c⁢t⁢a⁢n⁢g⁢l⁢e⁢s

(6) $$=2\,\left(\frac{1}{2}*\frac{t\,o\,t\,a\,l\,l\,e\,a\,f\,l\,e\,n\,g\,t\,h}{3}*a\,v\,e\,r\,a\,g\,e\,l\,e\,a\,f\,w\,i\,d\,t\,h\right)$$

The remaining two faces of the triangle (the sides of the top third of the leaf) were calculated using Eq. (8):

A⁢r⁢e⁢a⁢⁢o⁢f⁢⁢t⁢r⁢i⁢a⁢n⁢g⁢l⁢e⁢s⁢o⁢n⁢l⁢e⁢f⁢t⁢a⁢n⁢d⁢r⁢i⁢g⁢h⁢t⁢s⁢i⁢d⁢e

(7) $$=2\,\left(\frac{1}{2}*\frac{t\,o\,t\,a\,l\,l\,e\,a\,f\,l\,e\,n\,g\,t\,h}{3}*a\,v\,e\,r\,a\,g\,e\,l\,e\,a\,f\,t\,h\,i\,c\,k\,n\,e\,s\,s\right)$$

We summed all equations (Eqs. 4–8) to calculate total leaf surface area (cm^2^) as follows:

(8) L⁢e⁢a⁢f⁢s⁢u⁢r⁢f⁢a⁢c⁢e⁢a⁢r⁢e⁢a=E⁢q⁢. 4+E⁢q⁢. 5+E⁢q⁢. 6+E⁢q⁢. 7

Leaf mass per area (LMA; g m^–2^) was determined by dividing the dry mass by the leaf surface area.

### Leaf Anatomical Traits

**FIGURE 2:**
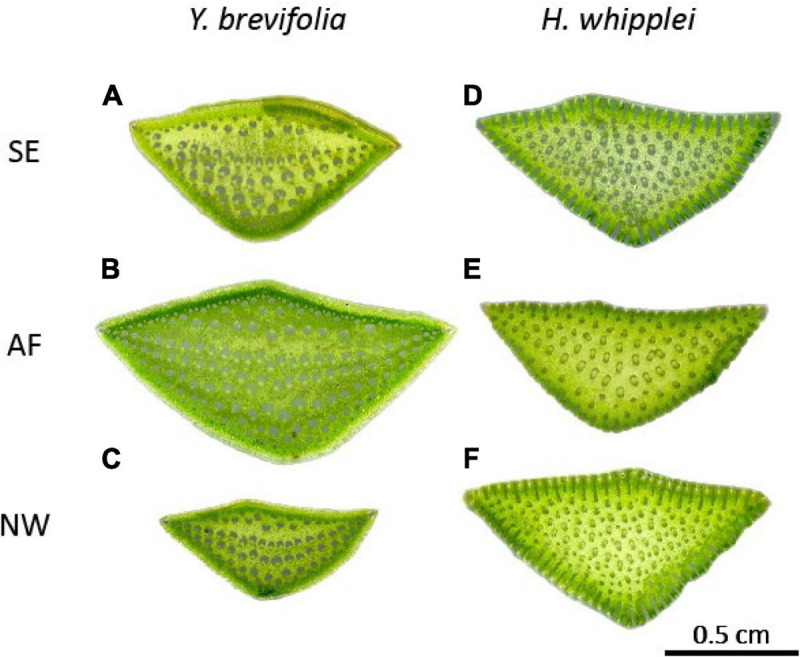
Leaf cross-sections of *Y. brevifolia* and *H. whipplei* from different microhabitats in the West Mojave Desert. *Y. bevifolia* **(A–C)** and *H. whipplei* **(D–F)** cross sections showing vascular bundles. SE, south-eastern slope; AF, alluvial fan; NW, north-western slope. *n* = 5 leaves per species per site.

Hand cross sections were made using double-edge razor blades from 5 leaves from 3 to 5 individuals, from both species and all three sites, and mounted to slides and scanned (flatbed scanner; Canon Scan Lide 90, 1,200 pixels per inch). The total number of vascular bundles and leaf cross-sectional area were measured using ImageJ. Each trait was measured twice by a different person to ensure accuracy and repeatability in measurements. No significant differences were found across the datasets obtained from the two different individuals (paired *t*-test, *p* > 0.05). Because of the 3-dimensional (3D) vein architecture that these species display (Figure 2), inter-vein density (IVD; μm) was measured using ImageJ (*n* = 5 leaves per species per site) by selecting a central vein and then measuring the distance to the nearest neighboring veins (number of neighboring veins ranging from 4 to 8 across samples and species). Inter-vein density was calculated as the average distance between the central vein and the neighboring veins. Full leaves were also scanned to measure leaf length using Image J. Because of the 3D arrangement of veins throughout the leaf length, it was not possible to get an accurate measurement of vein length per area (VLA) by clearing leaves. Instead, we cut and scanned (flatbed scanner; Canon Scan Lide 90, 1,200 pixels per inch) six leaf cross sections throughout the leaf length and counted the number of veins in each section. We also measured the cross-sectional area to determine the number of veins per area (mm^2^) as follows:

(9) $$N\,u\,m\,b\,e\,r\,o\,f\,v\,e\,i\,n\,s\,p\,e\,r\,m\,m^{2}=\frac{n\,u\,m\,b\,e\,r\,o\,f\,v\,e\,i\,n\,s\,p\,e\,r\,\sec t\,i\,o\,n}{c\,r\,o\,s\,s\,\sec t\,i\,o\,n\,a\,l\,a\,r\,e\,a}$$

The number of veins per mm^2^ was calculated for each of the six cross-sectional sections per leaf (*n* = 5). We then averaged values across sections and leaves and site for each species.

### Statistical Analyses

To test for physiological, morphological and anatomical variation across sites and species, we performed a *two-way* ANOVA followed by Tukey-Kramer *post hoc* test in R (R 3.5.3^2^). Prior to all statistical analyses, we log-transformed data to improve normality and heteroscedasticity (Sokal and Rohlf, 1995). We tested for a correlation between leaf width and stomatal conductance using organized least squares (in SMATR; Sokal and Rohlf, 1995; Warton et al., 2006), and for correlations between *g*_s_ and time of *g*_s_ measurement, and *g*_s_ and VPD using Pearson’s coefficient.

## Results

### Physiological Differences Across Sites and Species

**FIGURE 3:**
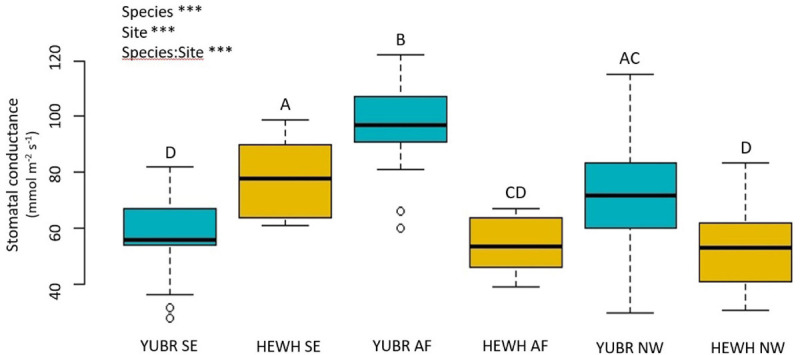
Stomatal conductance (*g*_s_) varied significantly across species and sites. Different letters denote statistically significant differences across species and sites (*p* < 0.05) by *two*-way ANOVA followed by a Tukey-Kramer *post hoc* test. YUBR = *Y. brevifolia*; HEWH = *H. whipplei*; SE, south-eastern slope; AF, alluvial fan; NW, north-western slope. *n* = 23–25 leaves per species per site. ***p < 0.001; ^*NS*^*p* > 0.05.

We found significant variation in stomatal conductance (*g*_s_) across species and sites. The *g*_s_ varied from 96.75 ± 2.9 mmol m^–2^ s^–1^ in the AF to 57.0 ± 2.7 mmol m^–2^ s^–1^ in the SE slope in *Y. brevifolia*, and from 78.3 ± 4.9 mmol m^–2^ s^–1^ to 53.2. ± 3.32 mmol m^–2^ s^–1^ in the SE and NW slope, respectively, in *H. whipplei* (Figure 3). We found a significant species-site interaction (*two-way* ANOVA; *p* < 6.29e-11), with *Y. brevifolia* growing at the AF having the highest *g*_s_ (Figure 3). *H. whipplei* individuals growing in the SE slope exhibited higher *g*_s_ than individuals in the AF and NW slope (Figure 3).

**FIGURE 4:**
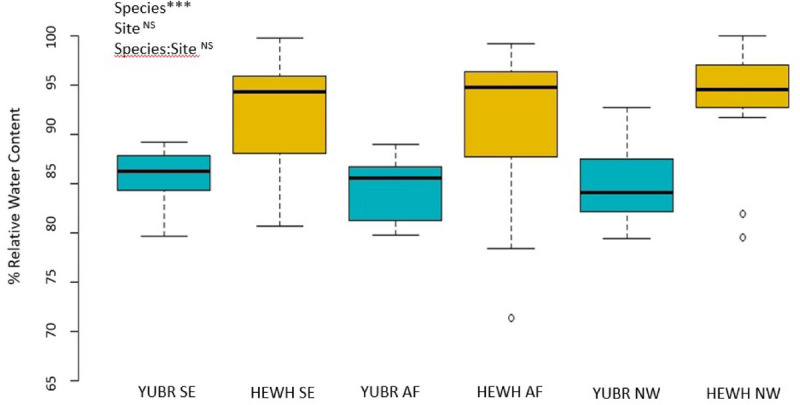
Relative water content (RWC) did not differ across sites. Results from *two-way* ANOVA are shown in the top left corner. YUBR = *Y. brevifolia*; HEWH = *H. whipplei*; SE, south-eastern slope; AF, alluvial fan; NW, north-western slope. *n* = 13–20 leaves per species per site. ***p < 0.001; ^*NS*^*p* > 0.05.

Relative water content did not differ across sites (*two-way* ANOVA; *p* = 0.262), and no species-site interaction was found (*two-way* ANOVA; *p* = 0.342). However, we found a significant difference in RWC between species (*two-way* ANOVA; *p* = 5.65e-8), with RWC in *Y. brevifolia* being on average 8% lower than in *H. whipplei* (Figure 4). No significant differences across sites or species were found in SWC (*two-way* ANOVA; *p* < 0.05).

The minimum epidermal conductance did not differ between species (*two-way* ANOVA; *p* = 0.2745), and no significant species-site interaction was found (*two-way* ANOVA; *p* = 0.0857). However, a significant site effect was observed with *g*_min_ being the highest in individuals growing at the SE slope (*two-way* ANOVA; *p* = 0.0433) (Supplementary Table S1).

### Morphological and Anatomical Differences Across Sites and Species

Leaf area was significantly different across species at each site (*two-way* ANOVA; *p* < 0.04), with *H. whipplei* leaves on average 2.2-fold larger than *Y. brevifolia* (average leaf area across sites = 98.9 ± 7.8 cm^2^ in *H. whipplei* and 45.3 ± 4.5 cm^2^ in *Y. breviolifa*; *two-way* ANOVA; *p* < 0.001; Supplementary Table S1). Leaf mass per area (LMA) displayed significant differences across species and species-site interaction (*two-way ANOVA*; *p* = 5.32e^–4^ − 1.93e^–6^, respectively), though these differences were driven by differences observed between the two species at the NW site, with *H. whipplei* displaying 1.7-fold higher LMA than *Y. brevifolia* (62.3 ± 5.2 vs. 36.1 ± 2.2 g m^–2^; Supplementary Table S1).

**FIGURE 5:**
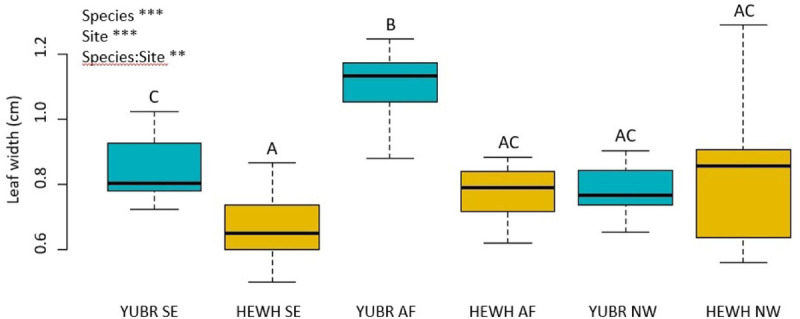
Leaf width varied across species, sites, and had significant species-site interactions. Different letters denote statistically significant differences (*p* < 0.05) by single-factor ANOVA followed by a Tukey-Kramer *post hoc* test. YUBR = *Y. brevifolia*; HEWH = *H. whipplei*; SE, south-eastern slope; AF, alluvial fan; NW, north-western slope. *n* = 9–11 leaves per species per site. ***p < 0.001; **p < 0.01.

**FIGURE 6:**
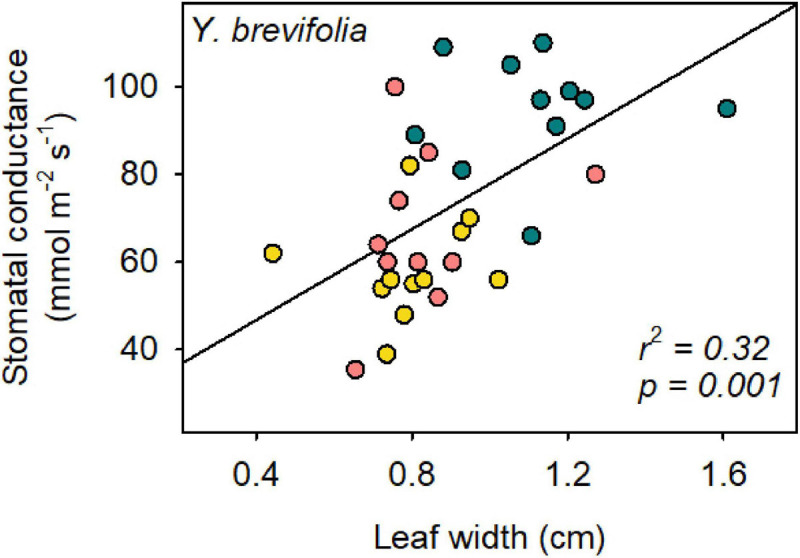
Leaf width correlates with stomatal conductance in *Y. brevifolia*. The *r*^2^- and *p*-value of the organized least square regression is provided in the panel. *Stomatal conductance* = 52 × *leaf width* + 26. Yellow, south-eastern slope; Blue, alluvial fan; Pink, north-western slope. *n* = 9–11 leaves per species per site.

Leaf width varied significantly across species, sites and in their interaction (*two-way* ANOVA; *p* = 0.003). Leaf width was larger on average in *Y. brevifolia*, and highest at the AF (Figure 5). However, no significant differences were found in leaf width across sites in *H. whipplei* (*two-way* ANOVA; *p* > 0.05, Figure 5). A significant positive correlation was found between leaf width and *Y. brevifolia* across sites (Figure 6), but not across individuals of *H. whipplei* (*p* > 0.05).

**FIGURE 7:**
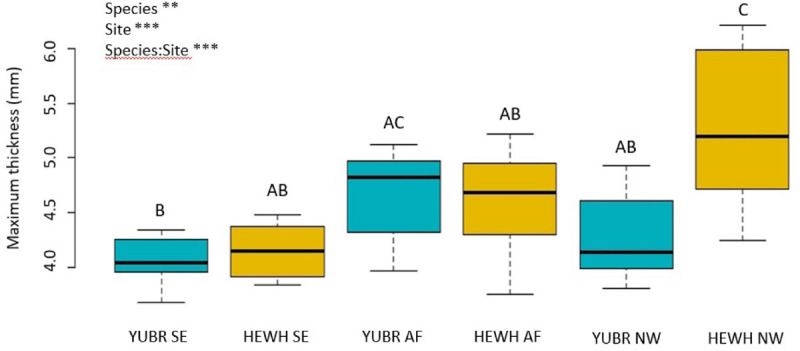
Maximum leaf thickness varied significantly across sites and species. Different letters denote statistically significant differences (*p* < 0.05) by single-factor ANOVA followed by a Tukey-Kramer *post hoc* test. YUBR = *Y. brevifolia*; HEWH = *H. whipplei*; SE, south-eastern slope; AF, alluvial fan; NW, north-western slope. *n* = 7–10 leaves per species per site. ***p < 0.001; **p < 0.01.

Leaf thickness was on average higher in *H. whipplei* than *Y. brevifolia* across sites (*two-way ANOVA*; *p* = 0.008; Figure 7), and a significant site and species-site interaction was found (*two-way ANOVA*; *p* < 0.001), with the highest value achieved by *H. whipplei* (5.25 ± 0.22 mm) in the NW slope, and smallest by *Y. brevifolia* (4.19 ± 0.15 mm) growing in the SE slope (Supplementary Table S1). No differences were found in leaf PLT across species, nor in site-species interaction (*two-way* ANOVA; *p* > 0.05). However, a significant site effect was found (*two-way* ANOVA; *p* = 0.003), with the NW slope showing 7–8% greater thickness shrinkage at midday than the AF and SE slope (Supplementary Table S1).

**FIGURE 8:**
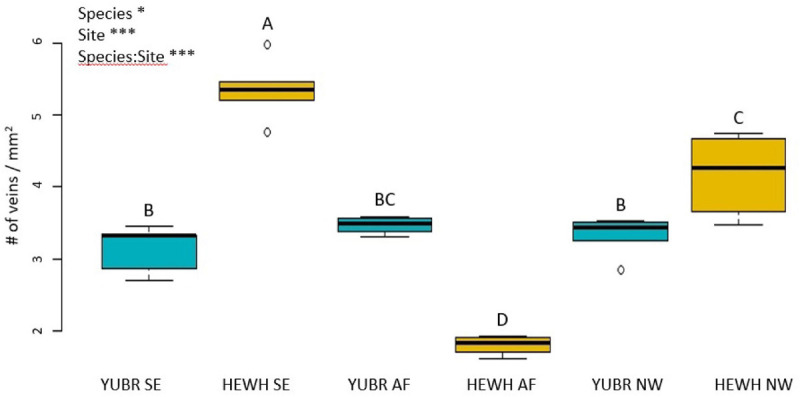
Number of veins per mm^2^ area varied across sites and had significant species-site interactions. Different letters denote statistically significant differences (*p* < 0.05) by single-factor ANOVA followed by a Tukey-Kramer *post hoc* test. YUBR = *Y. brevifolia*; HEWH = *H. whipplei*; SE, south-eastern slope; AF, alluvial fan; NW, north-western slope. *n* = 5 leaves per species per site. ***p < 0.001; *p < 0.05.

The number of veins per mm^2^ significantly varied across species and in their interaction with sites (*two-way* ANOVA; *p* < 0.0342; Figure 8). While *Y. brevifolia* showed no significant variation in the number of veins per mm^2^ across sites (3.02 ± 0.22 mm^2^ on average across sites), *H. whipplei* was highest in the SE slope (5.35 ± 0.2 mm^2^) and lowest in the AF (2.2 ± 0.4 mm^2^; Figure 8). No significant differences across sites or species were found in IVD (*two-way* ANOVA; *p* > 0.05) and averaged 307 ± 7.5 μm across sites and species.

## Discussion

Our results suggest that *H. whipplei* exhibits a higher water conservation strategy than *Y. brevifolia*, which performed better physiologically in the moister site. Indeed, we found variation in stomatal conductance (*g*_s_) across sites and species with highest values exhibited at the species’ preferred habitat (Haines, 1941; Cole et al., 2011). *Y. brevifolia* displayed higher *g*_s_ in the alluvial fan while *H. whipplei* displayed higher *g*_s_ in the shallow and exposed soils of the south-eastern slope. Notably, a recent meta-analysis of 54 case studies on the variation in gas exchange, carbon isotopes and water use efficiency showed that individuals were physiologically more efficient at their ecological optimum than when growing at their ecological range edge (Abeli et al., 2020). While our study did not directly measure the variation in stomatal conductance across the entire range of the *Y. brevifolia* and *H. whipplei*, our findings that species’ physiological maxima were found at the sites that best reflected their preferred habitat could suggest stomatal limitation as a potential driver of these succulent monocot population demographics in the Mojave deserts. Future studies are needed to test and confirm this hypothesis. Such studies would help improve our understanding of how population demographics are affected by climate change.

### Causes of Stomatal Conductance Variation Across Sites and Species

What is causing the observed variation in *g*_s_ across sites within species? Our results indicate that the *g*_s_ variation is not driven by differences in either leaf water status as relative water content was similar across sites within species or VPD. A previous study on a different *Yucca* species growing in Colorado (*Y. glauca*) found that the high VPD and temperature values in the summer at midday led to a strong decrease in stomatal conductance and photosynthetic rates (Roessler and Monson, 1985). In contrast, we found for our two species that at the end of winter/start of spring, VPD values were low on average even at midday (Table 1), and no negative correlations between *g*_s_ and time of measurement or VPD were found (Supplementary Figures S1, S2). Future studies should focus on investigating physiological variation throughout the year to identify possible seasonal patterns across species and sites. Additionally, long term physiological surveys are critical to understand the future trajectory of these responses especially under a changing climate.

The differences observed in *g*_s_ in our study are thus likely driven by underlying anatomical traits. While we were unable to quantify the stomatal density on neither leaves of *H. whipplei* nor *Y. brevifolia* due to the excessive epicuticular wax, we investigated variation in vein density which has been shown to correlate with stomatal density (Li et al., 2013). Water supply and demand are generally well correlated across diverse and closely related species (Scoffoni et al., 2016; Scoffoni and Sack, 2017). We found a coordination between the number of veins/mm^2^ of cross-sectional area and *g*_s_ in *H. whipplei*, where leaves of individuals growing on the south-eastern slopes had greater number of veins/mm^2^, associated to their higher *g*_s_. This higher vein density could help supply water to more numerous stomata, and thus increase stomatal conductance at given water potential. The higher vein density at this site might also enable greater sugar transport if the higher stomatal conductance enabled greater photosynthetic rates, as has been observed across diverse and closely related species (Brodribb et al., 2007; Scoffoni et al., 2016). Indeed, an increase in gas exchange driven by anatomical differentiation in shallow, rocky soils with poor water retention could help explain how individuals of this species are so successful at those sites across their ecological range.

Number of veins/mm^2^ could not explain the variation observed in *g*_s_ in *Y. brevifolia*, as our results showed that individuals did not differ significantly in that trait across sites. However, we found a strong correlation between leaf width and stomatal conductance across sites in *Y. brevifolia*. Indeed, leaf width was significantly higher in individuals growing at the alluvial fan in *Y. brevifolia* than in those growing on the slopes (Figure 5), matching the pattern observed for *g*_s_ in that species. Additionally, leaves of *Y. brevifolia* were significantly thicker in individuals growing at the AF compared to the SE slope. The thicker and wider leaves of *Y. brevifolia* at the AF could provide greater water storage, which could help maintain water supply to the transpiration stream and keep more stomata open at a given water potential.

Differences in other leaf morphological traits did not explain the variation observed in *g*_s_ at the three microhabitats for each species. Smaller leaves have thinner boundary layers which can lead to higher rates of transpiration and thus higher *g*_s_, all else being equal. *H. whipplei* leaves were larger than *Y. brevifolia* but no site effect was found, suggesting that the variation in *g*_s_ may not be explained by leaf surface area. Leaf percent loss thickness coordinates with the shrinkage inside of the leaf cells and tissues (Scoffoni et al., 2014). While in most species higher rates of leaf shrinkage can play a role in triggering stomatal closure (Scoffoni et al., 2014), in succulent species an opposite mechanism was proposed (Scoffoni et al., 2014). Indeed, because of the greater proportion of water storage cells in these species, shrinkage might occur without causing physiological damage to the plant (Ogburn and Edwards, 2013) and contribute to the plant’s ability to survive during drought. Here we found no significant differences in the percent loss of thickness in the field for neither of our species or in their interaction with sites. Our two species shrunk on average between 9.0 and 19.4% across sites, which is within the range of PLT values at turgor loss point observed across 14 diverse non succulent angiosperm species (average = 17.6 ± 2.43 %, ranging from 4.6% in *Raphiolepis indica* to 38% in *Lantana camara*; Scoffoni et al., 2014). These results suggest that our species were not suffering from drought stress at the time of measurement in the field. Notably, PLT was highest in individuals growing at the NW sites, which is also the site where individuals displayed greater *g*_min_. A greater loss of water through the epidermis could potentially help explain greater shrinkage at that site.

Notably, other organs, such as roots, may have a role in explaining the differences observed in gas exchange across species and sites. Both *Y. brevifolia* and *H. whipplei* have contractile roots which are specialized to contract longitudinally (North et al., 2008). *Y. brevifolia* roots appear to play a major role in the rapid uptake and delivery of water as the reduced endodermal suberization of proximal roots and shallow root growth was shown to facilitate water uptake and allow access to soil columns receiving maximum amounts of moisture (North and Baker, 2007). Future studies should focus on understanding plasticity in root hydraulics and morphology in these species and their impact on gas exchange. Finally, we collected data at the end of winter/start of spring to compare species across sites when water was least limiting and where they could thus exhibit highest rates of gas exchange. Future studies should focus on understanding the plasticity in gas exchange across sites throughout the year, and how much these vary in cumulative gas exchange throughout seasons using these different strategies.

### Ecological Implications Behind the Morphoanatomical Drivers of the Physiological Variation Across Sites

The ability of *H. whipplei* to “adjust” its vein density (and potentially its stomatal density) to enable higher gas exchange might allow *H. whipplei* to thrive in the most arid micro-habitats compared to *Y. brevifolia*. Previous studies have found that vein density is increased in species of arid habitats. Indeed, a meta-analysis of 796 species showed a strong positive correlation between vein density and the aridity index (Sack and Scoffoni, 2013). Additionally, previous work on *Eucalyptus* and *Corymbia* species has shown that species of arid habitats tend to overinvest in leaf venation to compensate for leaf morphological constraints on photosynthesis (de Boer et al., 2016). This increase in vein density in arid habitats is argued to help achieve high photosynthetic rates after a rare rain event (Maximov, 1931; Grubb, 1998; Scoffoni et al., 2011; Sack and Scoffoni, 2013). Across its relatively wide ecological range throughout southern California, *H. whipplei* thrives in shallow soil or rocky outcrops, which are characterized by poor water retention, and thus greater aridity. Additionally, we observed bundle sheath extensions in leaves of *H. whipplei* across all three sites, though these appear more pronounced in individuals growing in the slopes than in the alluvial fan (Figure 2). Bundle sheath extensions have been shown to help guide light to the mesophyll (Karabourniotis et al., 2000; Nikolopoulos et al., 2002; Kenzo et al., 2007). In the thick succulent leaves of *H. whipplei*, the more pronounced bundle sheath extensions in individuals growing at the slopes could help increase light availability to the deeper mesophyll of the leaves, and thus engage more numerous cells in photosynthesis. Indeed, we observed that many minor veins are found throughout the cross-section in this species (Figure 2), and not simply at the ring layer below the epidermis like other species displaying 3D vein architecture typically show (see section “Three-Dimensional Venation Architecture in Our Desert Succulents” in the discussion below). Future studies should quantify the plasticity in bundle sheath extensions, and major and minor vein architecture in this species, and how these relate to gas exchange and resistance to drought.

*Y. brevifolia* on the other hand are typically found on well-drained alluvial fans (Cole et al., 2011), where water is more readily accessible than on slopes after a rain event. Our results indicate that unlike *H. whipplei*, the greater stomatal conductance observed in *Y. brevifolia* at the alluvial fan site was not driven by differences in vein densities. There could be a disconnect between stomatal and vein densities across sites in this species, and the extra supply needed to meet the evaporative demand could be provided by either differences in xylem anatomies (greater xylem conduit sizes would provide more efficient flow), or mesophyll pathways which could improve outside-xylem hydraulic conductance (Buckley, 2015). Notably, leaves of *Y. brevifolia* were significantly thicker and wider at the AF sites, with a significant correlation between leaf width and stomatal conductance across sites in that species. This coordination suggests that greater succulence and water storage could help buffer changes in vapor pressure deficit throughout the day and maintain open stomata without causing a decrease in leaf water potential. Future work should investigate plasticity in xylem and outside-xylem hydraulic anatomy of *Y. breviolia* across micro-habitats.

### Three-Dimensional Venation Architecture in Our Desert Succulents

Our desert succulents display three-dimensional (3D) vein anatomy which, though extremely rare across species, has been hypothesized to have evolved in highly succulent leaves and other thick and/or rounded leaf species to help reduce the hydraulic path between veins and leaf cells that they supply, and thus improve photosynthesis (Ogburn and Edwards, 2013). Notably, the 3D venation that our two desert succulents display differs from the 3D arrangements that have been observed in other species. Indeed, to our knowledge, species from eight different plant families (Asparagaceae, Amaranthaceae, Asphodelaceae, Crassulaceae, Ellisochloa, Portulacineae, Proteaceae, and Molluginaceae) have previously been shown to display 3D venation with a midrib at the center of the leaf section, circled by a ring of higher order veins (Ogburn and Edwards, 2013; Heyduk et al., 2016), or one central layer of larger veins circled by a ring of higher order veins as observed in *Halothamnus auriculus* and in three other *Yucca* species (Kadereit et al., 2003; Heyduk et al., 2016). Here, our two Asparagaceae species display an extreme type of 3D venation (Figure 2): there are no central midrib or single layer of major veins circled by higher order veins, but rather major and minor veins are dispersed throughout the leaf cross-sections in 5–12 planes of veins in *H. whipplei* and 5–8 in *Y. brevifolia* (Figure 2). We note however that across sites, minor veins in *Y. brevifolia* appeared to be mainly located in the outer layers (near the epidermis) where the bulk photosynthesizing cells are found, whereas major veins were mainly distributed at the center of the leaf section and are surrounded by mostly water storage cells. However, in *H. whipplei* minor veins appear to be dispersed throughout the cross-section. *Y. brevifolia* has tighter and more forward-facing rosettes than *H. whipplei*, which could explain why veins appear more densely spaced on the sun exposed abaxial leaf surface (Figure 2). We hypothesize that the bundle sheath extensions in this species might allow light penetration deeper into the mesophyll and thus photosynthesis in cells throughout the cross-section. Future work quantifying the detailed leaf cross-sectional anatomy is needed to test these hypotheses.

## Data Availability Statement

The raw data supporting the conclusions of this article will be made available by the authors, without undue reservation.

## Author Contributions

AJ and CS wrote the manuscript with contributions from all authors. All authors contributed to the design of the study, data collection, and analysis.

## Conflict of Interest

The authors declare that the research was conducted in the absence of any commercial or financial relationships that could be construed as a potential conflict of interest.

## Footnotes

1 https://imagej.nih.gov/ij/

2 https://www.r-project.org/

## Supplementary Material

Supplementary Figure 1 Testing for an effect of time of measurement on stomatal conductance in *Y. brevifolia* **(A–C)** and *H. whipplei* **(D–F)** growing in different micro-habitats in the West Mojave Desert. SE, south-eastern slope, AF, alluvial fan, and NW, north-western slope. *n* = 23–25 leaves per species per site. The Pearson correlation coefficient and *p*-value are provided in each panel.

Supplementary Figure 2 Testing for an effect of vapor pressure deficit on stomatal conductance in *Y. brevifolia* **(A–C)** and *H. whipplei* **(D–F)** growing in different micro-habitats in the West Mojave Desert. SE, south-eastern slope, AF, alluvial fan, and NW, north-western slope. *n* = 23–25 leaves per species per site. The Pearson correlation coefficient and *p*-value are provided in each panel.

Supplementary Figure 3 Modeled leaf surface area.

Supplementary Table 1 Averages and raw data for the physiological and morpho-anatomical traits of the study.
