# Supplementary material for: Leaf Venation and Morphology Help Explain Physiological Variation in Yucca brevifolia and Hesperoyucca whipplei Across Microhabitats in the Mojave Desert, CA
Source: Front Plant Sci. 2021 Jan 8;11:578338. doi: 10.3389/fpls.2020.578338 (PMC7820123; doi:10.3389/fpls.2020.578338)
Supplement: Supplementary Figure 1 — Testing for an effect of time of measurement on stomatal conductance in Y. brevifolia (A–C) and H. whipplei (D–F) growing in different micro-habitats in the West Mojave Desert. SE, south-eastern slope, AF, alluvial fan, and NW, north-western slope. n = 23–25 leaves per species per site. The Pearson correlation coefficient and p-value are provided in each panel. [file Image_1.pdf]

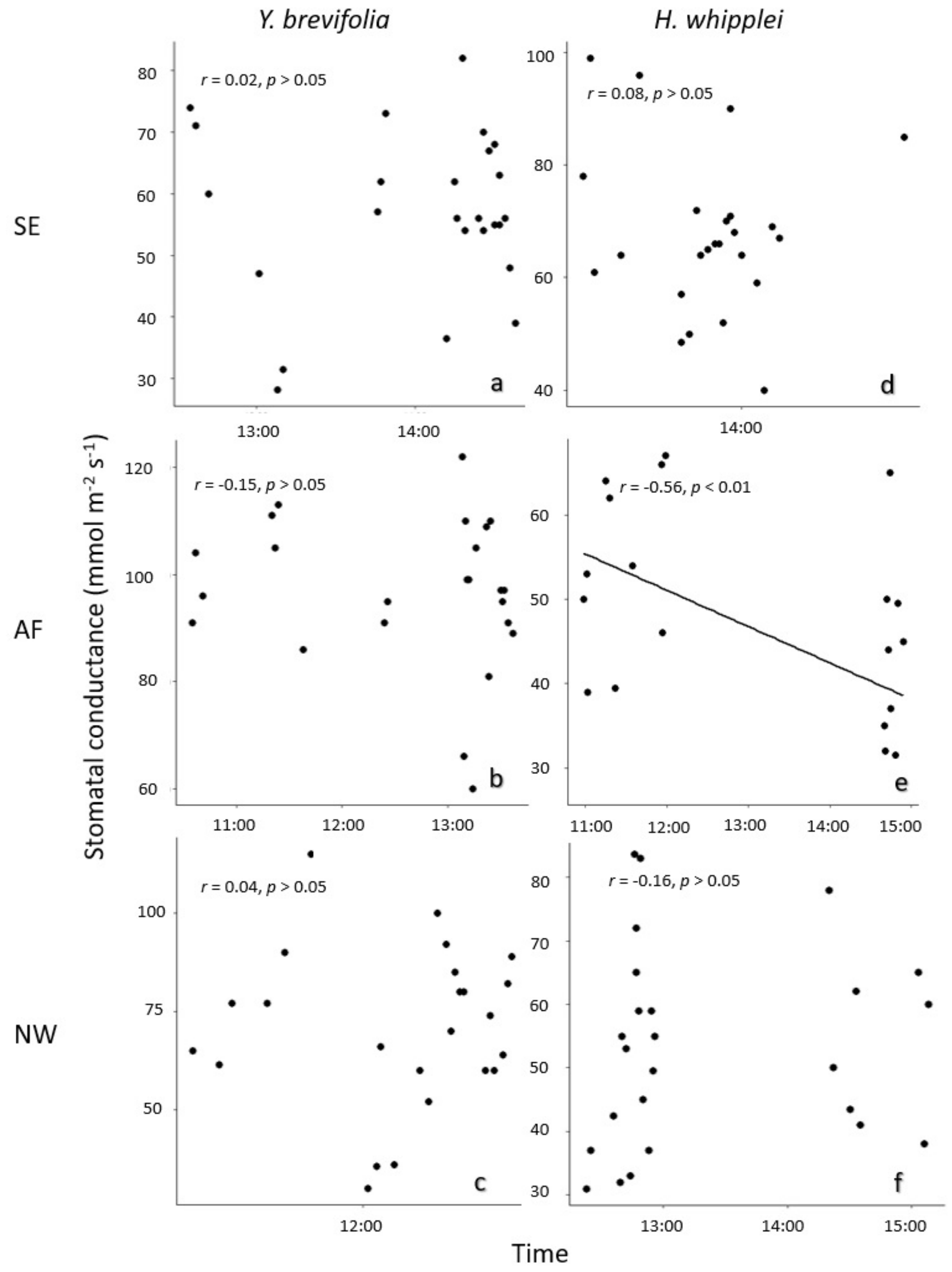

Figure S1. Testing for an effect of time of measurement on stomatal conductance in *Y. brevifolia* (a-c) and *H. whipplei* (d-f) growing in different micro-habitats in the West Mojave Desert. SE= south-eastern slope, AF= alluvial fan, and NW= north-western slope. *n* = 23–25 leaves per species per site.

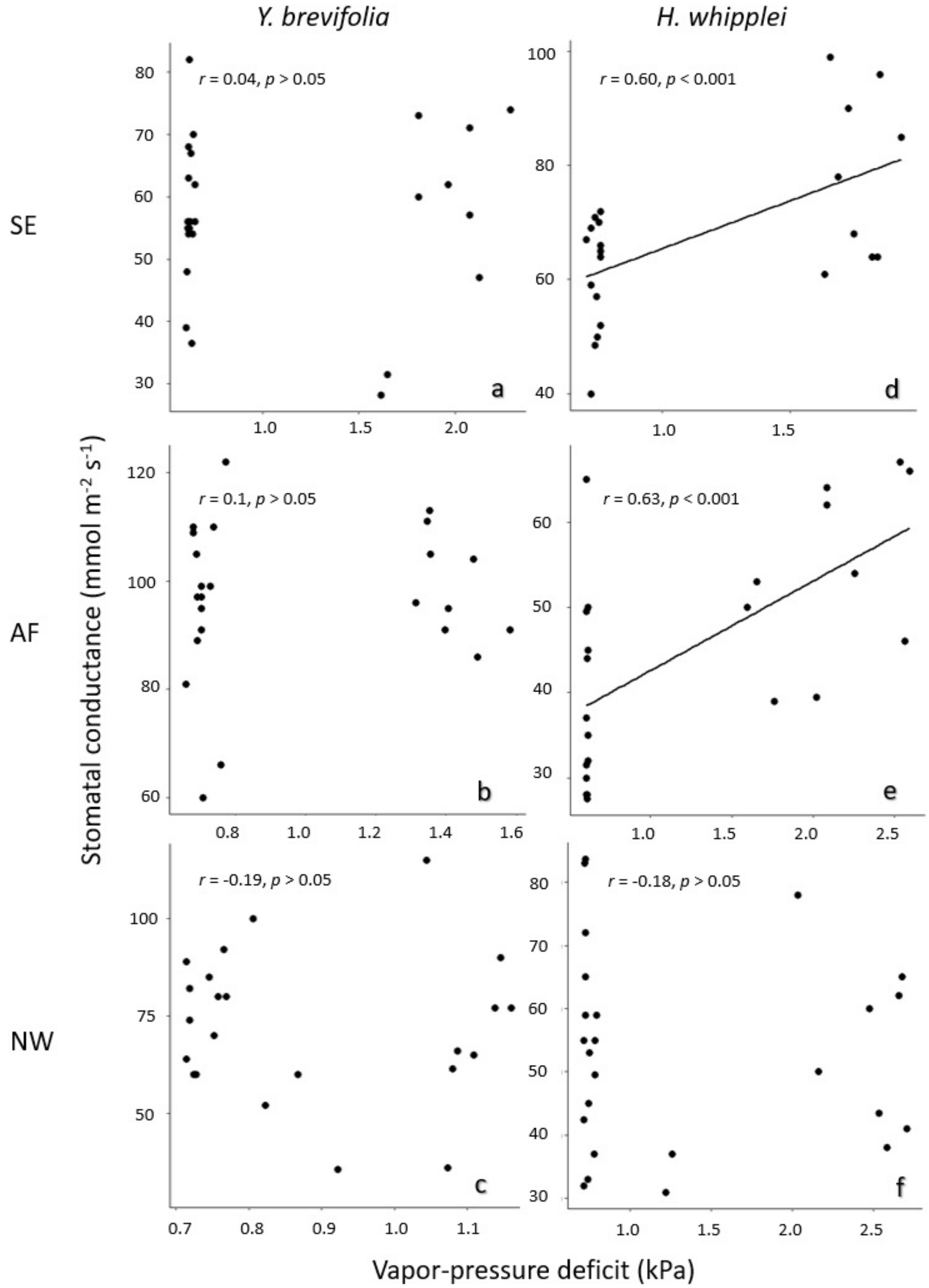

Figure S2. Testing for an effect of vapor pressure deficit on stomatal conductance in *Y. brevifolia* (a-c) and *H. whipplei* (d-f) growing in different micro-habitats in the West Mojave Desert. SE= south-eastern slope, AF= alluvial fan, and NW= north-western slope. n = 23–25 leaves per species per site.

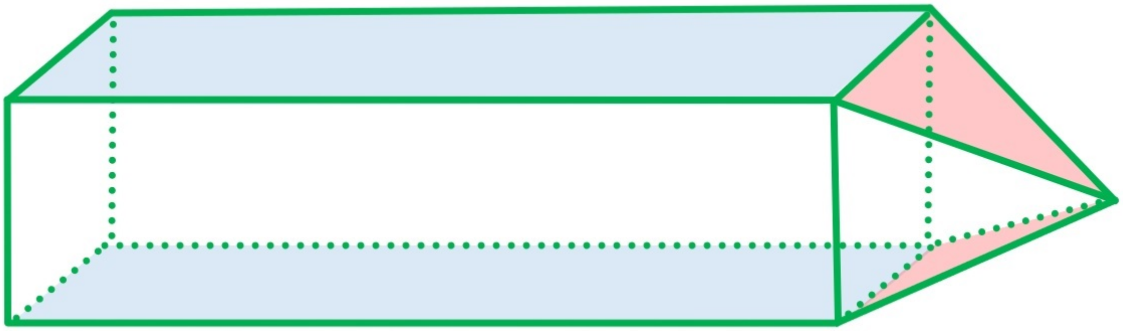

Figure S3. Modeled leaf surface area.
